# A Novel Strategy Facilitates Reference Gene Selection by RT-qPCR Analysis in Kidney Yang Deficiency Syndrome Mice Infected with the Influenza A (H1N1) Virus

**DOI:** 10.1155/2020/9075165

**Published:** 2020-04-25

**Authors:** Yepei Fu, Jia Yang, Shanshan Fan, Shaozhe Zhao, Syed Muhammad Ali Shah, Muhammad Akram, Rong Rong, Yong Yang

**Affiliations:** ^1^Shandong University of Traditional Chinese Medicine, Jinan, Shandong 250355, China; ^2^Department of Eastern Medicine, Directorate of Medical Sciences, Government College University, Faisalabad 38000, Pakistan; ^3^Shandong Provincial Collaborative Innovation Center for Antiviral Traditional Chinese Medicine, Jinan, Shandong 250355, China

## Abstract

In reverse transcription-quantitative polymerase chain reaction (RT-qPCR) studies, endogenous reference genes are routinely used to normalize the expression of target gene studies. In order to precisely evaluate the relative expression of genes in the cells of mice suffering from Kidney Yang Deficiency Syndrome (KYDS) in response to influenza A virus (IAV) H1N1 using RT-qPCR, it is crucial to identify reliable reference genes. In the present study, 15 candidate reference genes (Actb, *β*2m, Gapdh, Gusb, Tuba, Grcc10, Eif4h, Rnf187, Nedd8, Ywhae, 18S rRNA, Rpl13, Ubc, Rpl32, and Ppia) were investigated in lung cells from KYDS mice infected with IAV H1N1. NormFinder, BestKeeper, and GeNorm were used to assess the stability of reference genes. The results were authenticated over extended experimental settings by a group of 10 samples. In the present study, we explored a novel method using dual-gene combinations; the difference in gene expression between the model and normal control groups was statistically analyzed by an independent-samples *t*-test, and the difference in the mean value between the two groups was compared. A *P* value > 0.05 and the lowest absolute value of the difference indicated the optimal reference two-gene combination. Four additional host innate immune system-related genes (TLR3, TLR4, TLR7, and RIG-I) were analyzed together with the two treatment datasets to confirm the selected reference genes. Our results indicated that none of these 15 candidate reference genes can be used as reference gene individually for relative quantitative fluorescence PCR analysis; however, the combination of Grcc10 and Ppia, based on the process of calculating the higher *P* value and lower difference values between groups, was the best choice as a reference gene for the lung tissue samples in KYDS mice infected with IAV. This technique may be applied to promote the selection process of the optimal reference gene in other experiments.

## 1. Introduction

At present, reverse transcription-quantitative polymerase chain reaction (RT-qPCR) is considered to be a sensitive, accurate, highly specific, and fast measurement of gene expression with absolute or relative quantification [1]. The absolute quantification for a particular transcript gene copy number is calculated by the standard curve, and the relative quantification for a target gene may be measured for the relative expression compared with the reference gene [2]. As most researchers focus on analyzing the discrepant changes in gene mRNA expression levels, relative quantification has become a widely applied method for amplifying and detecting gene expression [3].

Gene expression analyses rely on the use of reference genes, which are internal controls for varied samples and different experimental settings. The selection of an appropriate reference gene is crucial and stable. However, a number of studies have demonstrated that commonly employed reference genes are unstable, as their transcription is closely regulated in various experimental settings and varies among different tissues. For example, Gapdh is known as a key enzyme in glycolysis in mammalian cellular organisms, and it is frequently used as a stable reference gene. However, its expression is not always constant, particularly in cancer, since the expression of Gapdh changes markedly in rapidly dividing and proliferating tumor cells, and improper reference gene selection may lead to inaccurate results in the target gene expression levels [4, 5]. Thus, potential reference genes must be evaluated for their stability by several algorithms, such as GeNorm [6], which are currently available as part of qBase Plus [7], NormFinder [8], and Best Keeper [9, 10].

In traditional Chinese medicine (TCM), Kidney Yang Deficiency Syndrome (KYDS) is one of the classical syndrome patterns and is characterized by a set of complaints, mainly including tenderness and weakness in the knees and lumbar regions, mental fatigue, difficulty in urination, enuresis, female sterility, reduction of auditory function, and dental abnormalities [11]. It is the most common syndrome in old age [12]. Influenza is a contagious viral infection that affects millions of individuals annually worldwide, and pandemic outbreaks may cause significant deaths in the population aged >65 years, and up to 90% mortality associated with influenza occur in this age group [13–16]. These susceptible individuals belong to high-risk people for influenza infection, who are mostly similar to the KYDS according to the principles of TCM [12, 17]. An epidemiological investigation was conducted in 2137 healthy elderly above 60 years old; the results showed that the incidence rate of kidney deficiency was 78.80% [17]; kidney deficiency syndrome prevalence in participants showed an increasing trend with increasing age and deteriorating health status [18]. Another epidemiological study on 2067 adults aged >60 years revealed that 45.33% suffered from KYDS, showing that KYDS is the predominant TCM syndrome in high-risk populations [19], and these elderly groups are at increased risk for serious flu complications. KYDS or influenza A virus (IAV) infection (H1N1) causes universal variations in gene expression, which represent the molecular basis of the biological effects of KYDS after IAV infection [20–27].

To explain the mechanisms behind KYDS following IAV infection in order to develop an effective treatment for this disease, understanding the changes that occur during gene transcription is crucial. Our previous study demonstrated that certain common reference genes (for example, Gapdh or Actb) were not stable in lung tissue from mice with KYDS after IAV infection, compared with normal control mice. In fact, commonly used algorithms have been applied to evaluate the most reliable reference gene. However, through *t*-test analysis between groups, the single or multiple reference genes analyzed by these general algorithms exhibit significant variations in expression between the normal and model groups (*P* < 0.05). Therefore, a novel method for reference gene selection is needed.

In the present study, 15 candidate reference genes (Actb, *β*2m, Gapdh, Gusb, Tuba, Grcc10, Eif4h, Rnf187, Nedd8, Ywhae, 18S rRNA, Rpl13, Ubc, Rpl32, and Ppia) were selected, which may be used as internal control genes for normalizing gene expression. The stability of expression for the abovementioned reference genes in mice suffering from KYDS infected with IAV (H1N1) and their mock-inoculated controls were assayed by means of three statistical algorithms (BestKeeper, NormFinder, and GeNorm) and compared in mouse lung tissue using *t*-tests. Our analysis may facilitate the selection of the most accurate reference genes for calculating target gene expression to assess the KYDS-IAV correlation. This new technique may prove useful for selecting the optimal reference gene in other experiments.

## 2. Materials and Methods

### 2.1. Animals

This study was performed in accordance with the recommendations of the National Institutes of Health. The protocol was approved by the Ethics Committee in Shandong University of Traditional Chinese Medicine. The animals were regularly monitored by the veterinarian staff. No pathogens were found. To minimize animal suffering during surgery, isoflurane gas was used for anesthesia. SPF male BALB/c mice (weight, 18-20 g) were purchased from the Jinan Pengyue Experimental Animal Breeding Company, Ltd. After 3 days of acclimatization, all mice were randomly assigned to two groups (*n* = 10 per group) and treated for 2 weeks: estradiol benzoate (8 mg/kg) was administered intraperitoneally for 7 days to establish the KYDS mouse model as previously described [28], while normal control mice were intraperitoneally injected with normal saline (NS). The physical signs in mice were observed, and rectal temperature and body weight were measured and recorded. All animals were kept under a 12 h dark/light cycle at 21-23°C. Environmental humidity was controlled at 60-70%. On the 8th day, KYDS mice (model group) were anaesthetized with isoflurane and intranasally inoculated with 20 *μ*l of viral suspension containing hemagglutination titer of 1 : 320 of influenza A/FM/1/47 (H1N1) virus, while the normal control group mice were inoculated with 20 *μ*l NS. The mice were then allowed access to food and water *ad libitum*.

### 2.2. Tissue Preparation

On the 14th day, according to the approved protocol, the mice were sacrificed. The lungs were isolated and weighed for the organ index calculation. After harvesting, small tissue samples were immediately submerged in RNAstore Reagent (Tiangen) at a dilution ratio of 1 : 10 (*w*/*v*) and then stored at 4°C.

### 2.3. Extraction of Total RNA

Total RNA was extracted from 10 to 20 mg tissue samples using RNAprep Pure Tissue Kit (Tiangen) according to the manufacturer's instructions. The yield and purity of RNA were determined with the Quawell 5000 spectrophotometer (Quawell Technology). For further analysis, RNA samples with an absorbance OD 260/280 ratio of 1.8-2.0 were used. The assessment of RNA integrity was performed by agarose gel electrophoresis.

### 2.4. Synthesis of Reverse-Transcribed cDNA

Using a 20 *μ*l reverse transcription system (FastQuant RT Kit with gDNase, Tiangen), first-strand cDNA was synthesized from 2 *μ*g total RNA with random hexamer oligonucleotide primers and incubated at 42°C for 3 min to protect total RNA from genomic DNA interference. Using FastQuant RT Enzyme, reverse transcription was performed at 42°C for 15 min. cDNA was stored at -20°C until further use. For qPCR analysis, each sample of cDNA was diluted 10 times with nuclease-free water.

### 2.5. qPCR

The Bio-Rad CFX Connect Real-Time System was used to conduct qPCR. A total of 20 *μ*l mixture containing 6 pmol each reverse and forward primer, 2 *μ*l cDNA, and 10 *μ*l 2Χ SuperReal PreMix Plus with SYBR Green I (Tiangen) was used for each reaction. The amplification conditions were as follows: 40 cycles at 95°C for 10 sec, 60°C for 32 sec, and 95°C for 15 min. Following amplification, the addition of a thermal denaturing cycle was performed to derive the dissociation curve of the PCR product to validate the specificity of the amplification. The measurement was performed three times for each sample.

For each primer, the efficiency of qPCR in exponential phase was measured employing standard curves (4-fold serial dilutions of pooled cDNA containing equal amounts of various sample sets). Mean quantification cycle (Cq) values of each serial dilution were plotted against the cDNA dilution factor logarithm, and the following equation was used to obtain its value: *E* = 10 (−1/slope) × 100. The determination of the linear dynamic range was performed by the correlation coefficient (*R*^2^), and the standard curve for each gene was created.

To obtain a standard for the SYBR assay, the IAV H1N1 pDZ-M plasmid was used as previously described [29] and quantified by spectrophotometric analysis. The copy numbers of the IAV H1N1 M gene were detected by the absolute quantitative method.

### 2.6. Primers

From “publically available” nucleotide sequences, 15 candidate reference genes were selected. The candidate genes were Gusb, Gapdh, Actb, *β*2m, Tuba, Grcc10, Eif4h, Rnf187, Nedd8, Ywhae, 18S rRNA, Rpl13, Ubc, Rpl32, and Ppia. Using NCBI BLAST (http://blast.ncbi.nlm.nih.gov/Blast.cgi), the primers were identified from designed nucleotide sequences. Primers were obtained from Beijing Liuhe BGI Technology Company with their certificates of analysis. The characteristics of the selected reference genes are listed in Table 1. The primers for authentication of selected reference genes were as follows: toll-like receptor (TLR)3: forward CAGGATACTTGATCTCGGCCTT and reverse TGGCCGCTGAGTTTTTGTTC; TLR4: forward CATGGATCAGAAACTCAGCAAAGTC and reverse CATGCCATGCCTTGTCTTCA; TLR7: forward CTGGAGTTCAGAGGCAACCATT and reverse GTTATCACCGGCTCTCCATAGAA; and retinoic acid-inducible gene-I (RIG-I): forward GCAGGTTACTGTGGACTTTGTG and reverse TGCCATTCTCCCTTTAGTGTCT. The primers for the IVA H1N1 M gene were as follows: forward 5′-CTGAGAAGCAGATACTGGGC-3′ and reverse 5′-CTGCATTGTCTCCGAAGAAAT-3′.

### 2.7. Data Analysis

Threshold setting and baseline correction were performed by automatic calculation with the CFX Maestro software (Bio-Rad Laboratories, Inc.). Using this software in single threshold mode, the value of Cq was determined.

The stability of the reference genes was assessed by these data using three common statistical programs (NormFinder, GeNorm, and BestKeeper). The Cq values were changed into relative quantities by using 2^-*Δ*Cq^ (ΔCq = the corresponding Cq value − minimum Cq value). Calculations of NormFinder and GeNorm depend upon these converted values; BestKeeper was used to analyze the raw Cq. The relative expression level of each target gene was calculated according to the 2^-*ΔΔ*Cq^ method for normalization of the gene to each potential reference gene [30]. An independent-samples *t*-test with SPSS statistical software was employed to assess the statistical significance of the differences in gene expression. *P* values < 0.05 were considered statistically significant. Microsoft Excel 2010 was used for variance analysis.

## 3. Results

### 3.1. Establishment of a KYDS-Virus Mouse Model

**Figure 1:**
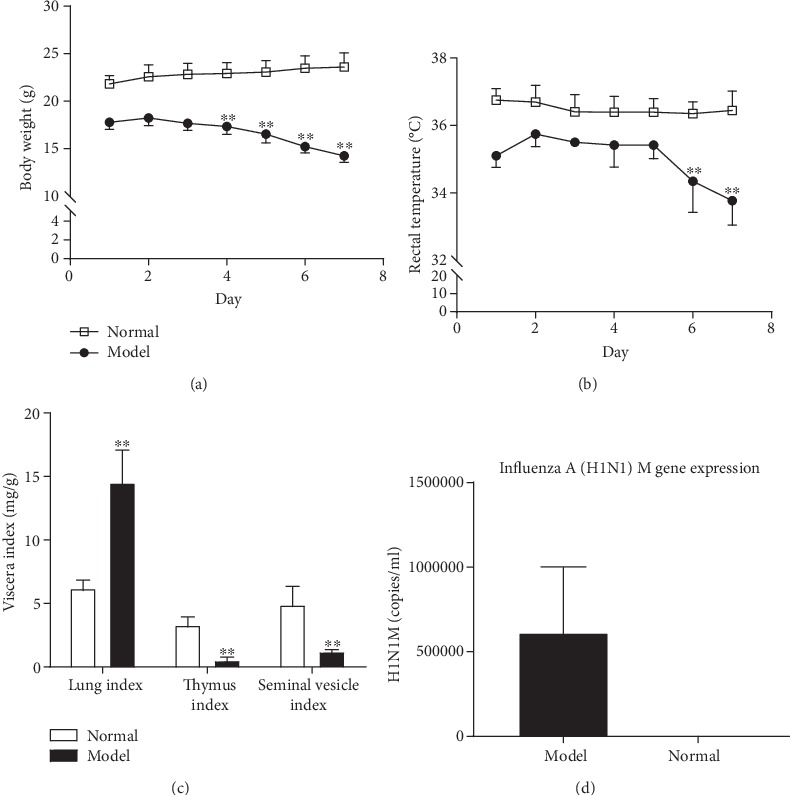
Assessment of the KYDS-virus model. (a) Whole body weight and (b) rectal temperature were measured after KYDS-virus model establishment for 7 days. (c) Visceral indices of different organs in mice. (d) Absolute quantity of influenza A virus (H1N1) M gene expression was calculated in the model group. ^∗∗^*P* < 0.01, compared with normal control; *n* = 10 in each group. KYDS: Kidney Yang Deficiency Syndrome.

After the KYDS mice were inoculated through the nose with IAV H1N1 or NS, the body weight and rectal temperature in the KYDS-virus group were found to be significantly lower compared with those in the normal control group from the 6^th^ to the 7^th^ day (*P* < 0.01) (Figures 1(a) and 1(b)). As shown in Figure 1(c), in comparison with the normal group, the model group thymus and seminal vesicle indexes decreased significantly (*P* < 0.01), whereas the lung index increased significantly (*P* < 0.01), clearly indicating that the model group lung tissue sustained inflammatory injury. IAV H1N1 mRNA expression was also detected. Total RNA was isolated from lung tissue and converted into cDNA. The copy numbers of H1N1 M gene RNA were >6.1 × 10^6^/ml, as determined using absolute quantification PCR by the standard curve (Figure 1(d)). These results demonstrated that the KYDS-virus model was successfully constructed.

### 3.2. Expression Profiles of Candidate Reference Genes

To accurately assess the patterns of the KYDS-virus expression studies, 15 candidate reference genes were finally selected on the basis of their common usage, as mentioned in the literature. Following qPCR analysis, each amplification primer set was tested for its performance. In all reactions, the amplification efficiency ranged from 91.0 to 97.2% and the standard curve correlation coefficients (*R*^2^) varied from 0.990 to 0.998 (Table 1). Strong correlation and very high efficiency were exhibited by all tested primer pairs. The amplification specificity of all transcripts was confirmed by melt curve analysis; as a single peak was produced, formation of primer dimers was not observed. Amplification was not observed in the absence of template (NTC control).

### 3.3. Analysis Using the BestKeeper, NormFinder, and GeNorm Algorithms

The GeNorm software was used for analysis of the expression stability of the 15 candidate reference genes. This software calculates gene expression stability (*M*) as the mean pairwise variation among all tested genes. The reference gene that yields the most similar results in all samples, regardless of the experimental circumstances, is considered the best reference gene. The reference gene with the smallest value of *M* was considered the most stable gene.

**Figure 2:**
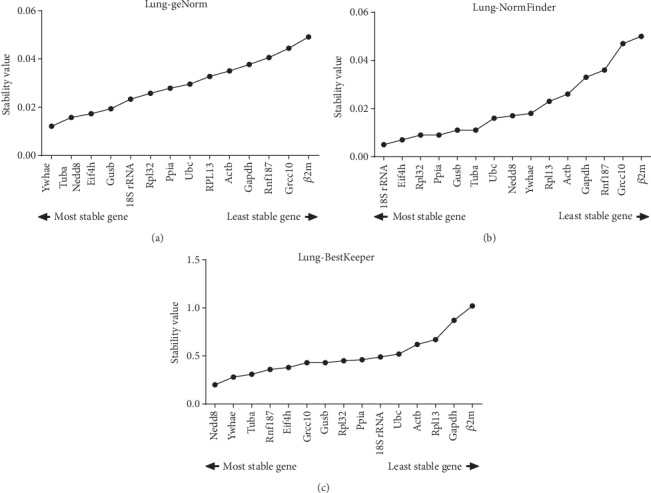
Mean expression stability (*M*) evaluated using GeNorm and NormFinder and mean expression stability standard deviation [SD (±CP)] evaluated using BestKeeper. *M* values and SD values rankings are indicated for mouse lung tissue under different conditions. (a) GeNorm, (b) NormFinder, and (c) BestKeeper.

A sample with *M* value < 1.5 is usually considered a stably expressed gene (Figure 2(a)). For lung tissue, Ywhae and Tuba are the two most stable reference genes, with the lowest *M* values (*M* = 0.012), whereas the *M* values of Grcc10 and *β*2m (*M* = 0.044 and 0.049, respectively) were the highest, indicating that those genes were the most variably expressed.

NormFinder is used for the calculation of the gene stability index (*M*). The lowest *M* values have been observed in the most stably expressed genes (Figure 2(b)). The most stable gene was 18S rRNA, whereas the best gene combination was Eif4h and Rpl32 (Table 2).

BestKeeper software was employed as an expression standard of reference genes with the ranking of the standard deviation [SD (±CP)]. The most stable reference genes have the lowest SD values. The acceptable range of variation of SD values is <1 (Figure 2(c)). Nedd8 was ranked as the most stably expressed gene in lung tissue, with the lowest SD value by BestKeeper.

### 3.4. Independent-Samples *t*-Test with Combination of Pair Candidate Reference Genes

We analyzed the combination of pair candidate reference genes in the normal and model groups with the independent-samples *t*-test, and 15 combinations of *P* values > 0.05 were identified (Table 3). Subsequently, for these potential combinations of reference genes, the absolute difference in mean Cq values between the two groups was calculated, and the smallest absolute difference value indicated that the combination of the genes was the optimal stable candidate reference gene. Based on these results (Table 4), the best combination of genes was Grcc10 and Ppia.

### 3.5. Validation of the Selected Reference Genes

**Figure 3:**
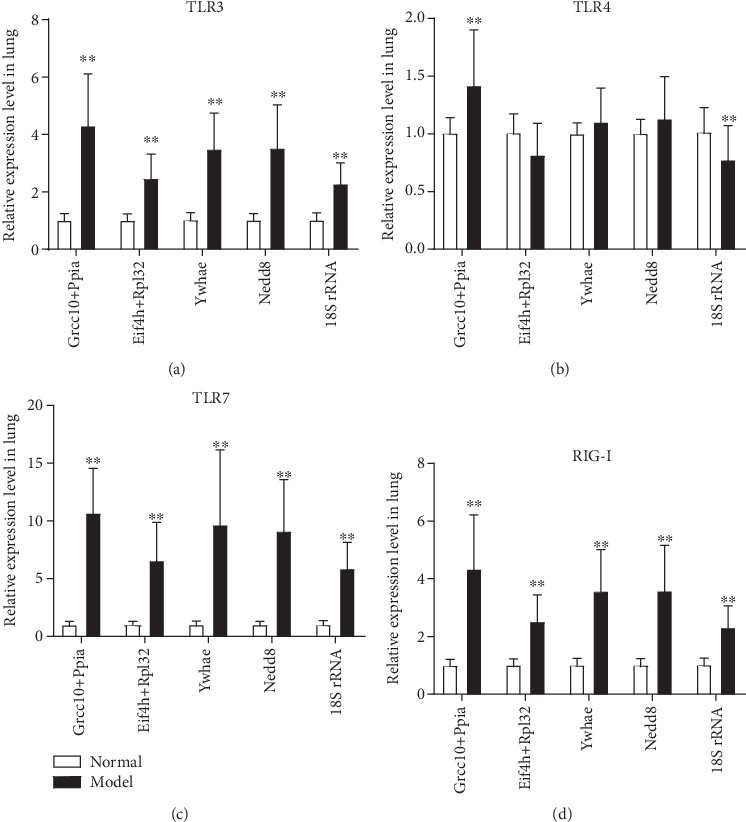
Relative expression levels of toll-like receptor (TLR)3, TLR4, TLR7, and retinoic acid-inducible gene-I (RIG-I) in lung tissues from normal and model mice were measured by reverse transcription-quantitative polymerase chain reaction and normalized to the indicated reference gene. The fold changes of model (KYDS-virus) mice are indicated next to the bars. The expression levels of normal mice were set at a relative expression of 1 (^∗∗^*P* < 0.01 vs. the normal group; *n* = 10 in each group, mean ± standard deviation). KYDS: Kidney Yang Deficiency Syndrome.

For validation of the reliability of selected reference genes, the changes in these gene expressions in the KYDS-virus samples were investigated. An analysis of the relative expression level of target genes was performed. The data confirmed the reliability of the recommended reference genes in mouse lung tissue. The expression of TLR3, TLR4, TLR7, and cytosolic RIG-I, which play important roles in the host's innate immune response, was examined. The interferon regulatory transcription factor is triggered by this interaction, and NF-*κ*B is activated, which affects the expression of numerous chemokines and proinflammatory cytokines [31]. Thus, the expression of TLR3, TLR4, TLR7, and RIG-I was investigated in mouse lung tissue (Figure 3).

By using the recommended reference genes from the abovementioned analysis, the combination of Grcc10 and Ppia (independent-samples *t*-test), and the combination of Eif4h and Rpl32 (NormFinder), Ywhae (GeNorm), Nedd8 (BestKeeper), and 18S rRNA (NormFinder), we observed a significant increase of TLR3, TLR7, and RIG-I expression in the model group compared with the normal group (Figures 3(a), 3(c), and 3(d), *P* < 0.01). But the increased levels were different among these recommended reference genes, and the combination of Grcc10 and Ppia had the highest relative gene expression. For TLR4 study, the expressions of these candidate reference genes were very different, and only the combination of Grcc10 and Ppia and the single recommended gene 18S rRNA expression exhibited obvious changes in the model group compared with the normal group (Figure 3(b), *P* < 0.01). The combination of Grcc10 and Ppia exerted an upregulating effect; however, 18S rRNA exerted a downregulating effect on TLR4 gene expression. These results demonstrate the importance of selecting suitable reference genes for normalization to obtain reliable results in gene expression studies for the KYDS-virus treatment.

### 3.6. Diagram of Strategy for Screening Reference Gene

**Figure 4:**
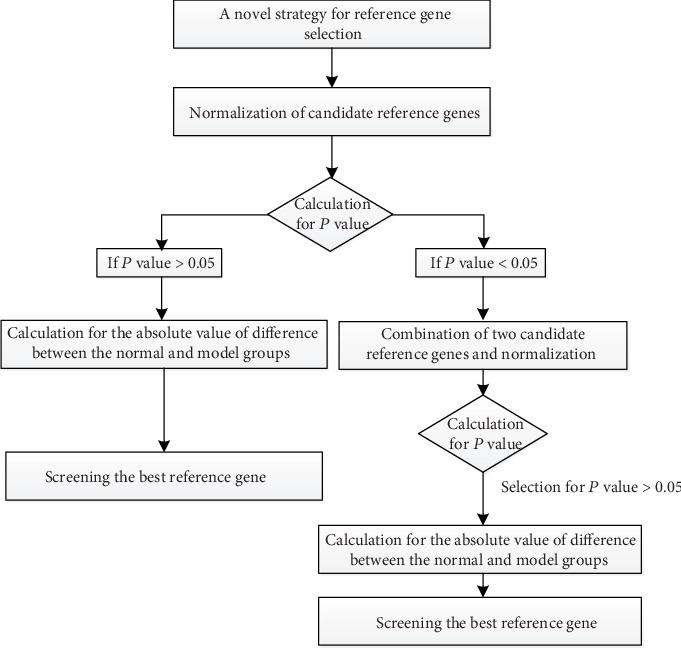
Diagram of strategy for screening reference gene.

To summarize, the novel strategy for screening reference gene is mainly based on the independent-samples *t*-test and comparison of absolute value of difference for different groups. This novel strategy could determine the best single reference gene or the best combination of two genes (Figure 4).

## 4. Discussion

In molecular biological research, the most frequently used strategy to study genes is gene expression analysis. qPCR is generally employed to study relative gene expression due to its versatility and accuracy. The results obtained from qPCR are strongly affected by the stability of the reference gene selected to normalize the gene expression data; therefore, it is crucial to select stably expressed genes as internal references.

We therefore screened suitable genes for data normalization in KYDS-virus mouse lung tissue. The expression stability of 15 reference genes (Actb, *β*2m, Gapdh, Gusb, Tuba, Grcc10, Eif4h, Rnf187, Nedd8, Ywhae, 18S rRNA, Rpl13, Ubc, Rpl32, and Ppia) was analyzed using three very common software packages: GeNorm, NormFinder, and BestKeeper. These statistical algorithms have been employed to select and validate reference genes for RT-qPCR data normalization across numerous tissues, species, and different treatments. Grcc10, Eif4h, Rnf187, Nedd8, and Ywhae are all considered consistently and highly expressed genes that can be used as reference controls in expression profiling analysis by RNA-seq [32], particularly in mouse lung tissue. Additionally, two widely used reference genes (Gapdh and Actb) were selected. However, in numerous cases, the expression of these genes has been found to be variable in various tissues, cells, and experimental conditions, as discussed by Suzuki et al. [33]. In particular, cellular macromolecule synthesis (shut-off) is inhibited by influenza virus infection and causes global gene expression changes affecting the mRNA levels of several genes [34, 35].

Our previous work has found that traditionally used reference genes, for example, Gapdh or Actb, were not stable in lung tissue in KYDS after IAV infection compared with normal control mice (data not shown). It has also been reported that influenza virus infection strongly affects the expression levels of the Actb and Gapdh reference genes, and they are therefore not reliable [36].

**Figure 5:**
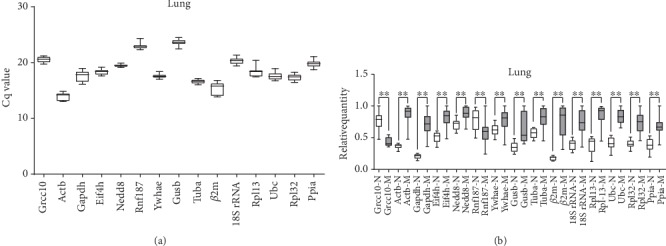
Distribution of quantification cycle (Cq) value and relative quantity for candidate reference genes. Box-whisker plot showing raw Cq values (a) and relative quantity (b) distribution of each reference gene in lung tissue. Boxes represent the quartiles with medians, whereas the maximum and minimum values are represented by whiskers.

Ywhae and Tuba were ranked as the most stable reference genes by GeNorm; 18S rRNA and the combination of Eif4h and Rpl32 were selected by NormFinder; and Nedd8 was recommended by BestKeeper. Although these software types are based on different algorithms, it was revealed that a single candidate reference gene was not suitable for relative gene expression (Figure 5(b)). We also proved that the combination of Eif4h and Rpl32 by NormFinder was not suitable as a reliable recommended reference gene. In the present study, a new strategy facilitating reference gene selection by RT-qPCR analysis in KYDS mice infected with IAV H1N1 was explored. An independent-samples *t*-test was used to evaluate the combination of candidate reference gene pairs between the normal and model groups, and 15 gene combinations were selected (*P* > 0.05). Subsequently, the absolute difference in mean Cq values for these potential combinations of reference genes was calculated in the two groups, and the smallest absolute difference value indicated that the combination of the genes was the best stable candidate reference gene. In view of these results (Table 4), the best combination of genes was Grcc10 and Ppia.

Under various experimental conditions, unreliable conclusions have been reached due to the instability of the reference gene; it is therefore crucial to verify reference gene stability for each normalizing experiment that attempts to show little differences in mRNA abundance. By using the different recommended reference genes from different algorithms, we observed similar changes in TLR3, TLR7, and RIG-I after KYDS-virus model establishment. The outcomes normalized with different reference genes can be detrimental, as it can suggest that there is no change or even that the change is in the opposite direction with the TLR4 target gene. In the present study, we investigated this phenomenon when the data were normalized with a single or paired reference gene. In different treatment groups, the target gene expression could appear to be markedly different, yielding biased results. Our data demonstrated that it is crucial to apply validated reference genes and to study their expression stability when employed for normalization of RT-qPCR data.

In conclusion, to the best of our knowledge, this is the first study especially planned to assess the effects of KYDS-virus on mouse lung tissue using a set of reference genes for gene expression normalization with the help of RT-qPCR in a BALB/c mouse model. For statistical analysis, NormFinder, GeNorm, BestKeeper, and independent-samples *t*-test were used. The four algorithms yielded different results with respect to the ranking of these candidate reference genes, indicating the importance of using more than one software type to achieve the best result. It is also concluded that, among 15 candidate reference genes investigated, any individual reference gene is not suitable for KYDS-virus mice. The combination of reference genes is necessary, and the combination of Grcc10 and Ppia was found to be the most stable for the KYDS-virus mouse model. Validation of the selected reference genes with target genes (TLR3, TLR4, TLR7, and RIG-I) under certain experimental conditions was also performed. Selecting suitable reference genes is crucial for obtaining reliable results in gene expression research. The present study provides a basis for reference gene selection and practical strategies for future gene expression studies of KYDS combined with IAV infection. This method may be generally applied to promote the selection of the optimal reference gene in other experiments.

## Figures and Tables

**Table 1:** Candidate reference genes and characteristics of RT-qPCR primers.

Gene symbol	Gene name	Accession no.	Primer sequence	Amplicon size	Efficiency (%)	*R* ^2^
Actb	Beta-actin	NM_007393.5	F: CCTTCTTGGGTATGGAATCCTGT R: ACTGTGTTGGCATAGAGGTCTTTAC	101	95.9	0.990
*β*2m	Beta-2 microglobulin	NM_009735.3	F: CATGGCTCGCTCGGTGAC R: CAGTTCAGTATGTTCGGCTTCC	135	96.8	0.995
Tuba	Alpha-tubulin	NM_011653.2	F: TGTCCTGGACAGGATTCGC R: CTCCATCAGCAGGGAGGTG	115	95.0	0.997
Gusb	Beta-glucuronidase	NM_010368.1	F: CCGACCTCTCGAACAACCG R: GCTTCCCGTTCATACCACACC	169	96.8	0.993
Gapdh	Glyceraldehyde-3-phosphate dehydrogenase	NM_008084.3	F: TGCACCACCAACTGCTTAG R: GGATGCAGGGATGATGTTC	177	94.2	0.996
Grcc10	Gene rich cluster, C10 gene	NM_013535.1	F: GCGGAGGTGATTCAAGCG R: TGACCAGGCGGGCAAACT	196	96	0.995
Eif4h	Eukaryotic translation initiation factor 4H	NM_033561.2	F: CCTTGGCTCGGGATTGTC R: TCCGCATTGGAGATGGATTA	198	97.2	0.998
Rnf187	Ring finger protein 187	NM_022423.2	F: CTGGCACCACCCTTCATC R: ACAAGCCCGAGCACATTC	155	95.1	0.991
Ywhae	Tyrosine 3-monooxygenase/tryptophan 5-monooxygenase activation protein	NM_009536.4	F: CCCATTCGTTTAGGTCTT R: TCCACAGCGTCAGGTTAT	193	93.2	0.994
Nedd8	Neural precursor cell expressed, developmentally downregulated gene 8	NM_008683.3	F: TGGGAAGGAGATTGAGATAG R: TTGCTTGCCACTGTAGATG	121	94	0.995
18S rRNA	18S ribosomal RNA	NR_003278.2	F: TTGACGGAAGGGCACCACCAG R: GCACCACCACCCACGGAATCG	130	91.7	0.996
Rpl13	Ribosomal protein L-13	NM_016738.5	F: GTACGCTGTGAAGGCATCAA R: ATCCCATCCAACACCTTGAG	135	91	0.997
Ubc	Ubiquitin C	NM_019639	F: GCCCAGTGTTACCACCAAGA R: CCCATCACACCCAAGAACA	104	96.3	0.996
Rpl32	Ribosomal protein L32	NM_172086.2	F: GAACTGGCGGAAACCCA R: GGATCTGGCCCTTGAACCTT	63	96.2	0.995
Ppia	Peptide acyl preserved ammonia acyl isomerase A	NM_008907.1	F: CGCTTGCTGCAGCCATGGTC R: CAGCTCGAAGGAGACGCGGC	86	96.3	0.990

The correlation coefficients (*R*^2^) of the standard curve, efficiency of each pair of primers, length of the RT-qPCR transcripts, and primer sequences are indicated. RT-qPCR: reverse transcription-quantitative polymerase chain reaction.

**Table 2:** NormFinder calculation results.

Variables	Results
Best gene	18S rRNA
Stability value	0.005
Best combination of two genes	Eif4h and Rpl32
Stability value for the best combination of two genes	0.004

**Table 3:** *P* values of the independent-samples *t*-test for the 15 candidate reference gene combinations.

	Grcc10	Actb	Gapdh	Eif4h	Nedd8	Rnf187	Ywhae	Gusb	Tuba	*β*2m	18S rRNA	Rpl13	Ubc	Rpl32	Ppia
Grcc10		0.043	0.001	0.538	0.010	0.000	0.027	0.821	0.113	0.001	0.801	0.211	0.258	0.803	0.986
Actb	0.043		0.000	0.000	0.000	0.009	0.001	0.000	0.001	0.000	0.000	0.000	0.000	0.000	0.000
Gapdh	0.001	0.000		0.000	0.000	0.001	0.000	0.000	0.000	0.000	0.000	0.000	0.000	0.000	0.000
Eif4h	0.538	0.000	0.000		0.000	0.293	0.003	0.000	0.001	0.000	0.000	0.000	0.000	0.000	0.000
Nedd8	0.010	0.000	0.000	0.000		0.686	0.012	0.002	0.005	0.000	0.000	0.000	0.000	0.000	0.001
Rnf187	0.000	0.009	0.001	0.293	0.686		0.810	0.266	0.712	0.001	0.059	0.023	0.010	0.045	0.145
Ywhae	0.027	0.001	0.000	0.003	0.012	0.810		0.003	0.006	0.000	0.001	0.001	0.000	0.000	0.000
Gusb	0.821	0.000	0.000	0.000	0.002	0.266	0.003		0.001	0.000	0.000	0.000	0.000	0.000	0.000
Tuba	0.113	0.001	0.000	0.001	0.005	0.712	0.006	0.001		0.000	0.000	0.000	0.000	0.000	0.000
*β*2m	0.001	0.000	0.000	0.000	0.000	0.001	0.000	0.000	0.000		0.000	0.000	0.000	0.000	0.000
18S rRNA	0.801	0.000	0.000	0.000	0.000	0.059	0.001	0.000	0.000	0.000		0.000	0.000	0.000	0.000
Rpl13	0.211	0.000	0.000	0.000	0.000	0.023	0.001	0.000	0.000	0.000	0.000		0.000	0.000	0.000
Ubc	0.258	0.000	0.000	0.000	0.000	0.010	0.000	0.000	0.000	0.000	0.000	0.000		0.000	0.000
Rpl32	0.803	0.000	0.000	0.000	0.000	0.045	0.000	0.000	0.000	0.000	0.000	0.000	0.000		0.000
Ppia	0.986	0.000	0.000	0.000	0.001	0.145	0.000	0.000	0.000	0.000	0.000	0.000	0.000	0.000	

**Table 4:** Screening results of 15 reference gene combinations with *P* values, standard deviation, and absolute values of difference.

Rank	Combination	*P* value	Standard deviation	Absolute value of difference
1	Grcc10+Ppia	0.986	0.156	0.009
2	Grcc10+Gusb	0.821	0.221	0.0096
3	Rnf187+Tuba	0.712	0.228	0.018
4	Grcc10+Rpl32	0.803	0.133	0.019
5	Grcc10+18S rRNA	0.801	0.180	0.0275
6	Grcc10+Eif4h	0.538	0.159	0.061
7	Nedd8+Rnf187	0.686	0.187	0.074
8	Rnf187+Ywhae	0.810	0.226	0.075
9	Grcc10+Ubc	0.258	0.144	0.113
10	Eif4h+Rnf187	0.293	0.228	0.1235
11	Grcc10+Tuba	0.113	0.154	0.1665
12	Rnf187+Gusb	0.266	0.325	0.175
13	Rnf187+Ppia	0.145	0.210	0.1935
14	Rnf187+18S rRNA	0.059	0.252	0.212
15	Grcc10+Rpl13	0.211	0.232	0.237

## Data Availability

The data used to support the findings of this study are available from the corresponding author upon request.

## Supplementary Materials

Supplementary Materials Gapdh description: these are RT-qPCR raw data of Gapdh genes. The normal group and the KYDS model group both have good melt peak curves, but they have obvious differences in Ct values more than two times. The Ct values of Gapdh description: these are Ct values of Gapdh genes. The average of Ct values in the normal group is 21.90; the average of Ct values in the model group is 19.89. The difference value between the two groups is 2.02. The Ct values of 5 reference gene description: this excel file includes Ct values of the normal and model groups from 5 reference genes (Gusb, Gapdh, Actb, *β*2m, and Tuba). They were analyzed with statistical method by Student's *t*-test; all the paired groups have a significant difference (*P* < 0.01).
